# A Case of Infantile Hepatic Hemangioendothelioma/Hemangioma at Maharaj Nakorn Chiang Mai Hospital

**DOI:** 10.7759/cureus.25240

**Published:** 2022-05-23

**Authors:** Komson Wannasai, Jongkolnee Settakorn, Pannee Visrutaratna, Lalita Sathitsamitphong, Jiraporn Khorana, Supanat Waroonkun

**Affiliations:** 1 Department of Pathology, Faculty of Medicine, Chiang Mai University, Chiang Mai, THA; 2 Department of Radiology, Faculty of Medicine, Chiang Mai University, Chiang Mai, THA; 3 Department of Pediatrics, Faculty of Medicine, Chiang Mai University, Chiang Mai, THA; 4 Department of Surgery, Faculty of Medicine, Chiang Mai University, Chiang Mai, THA; 5 Department of Medicine, Faculty of Medicine, Chiang Mai University, Chiang Mai, THA

**Keywords:** hepatic tumor, hepatectomy, case report, hepatic hemangioendothelioma, infantile

## Abstract

Infantile hepatic hemangioendothelioma/hemangioma is the most common benign hepatic vascular tumor of infancy, comprising approximately 1% of all childhood tumors. The tumor can present during the fetal or neonatal period as a hepatic mass. Common presentations include abdominal distension and a palpable hepatic mass. Clinico-radio-pathological correlation is essential for a definite diagnosis. Frequent complications such as congestive heart failure, thrombocytopenia, anemia, and Kasabach-Merritt syndrome should be investigated. Chemotherapy has been reported as an effective treatment option. Surgical resection has an essential role for symptomatic patients with medical treatment failure or other certain conditions such as refusal to take medication. Furthermore, prenatal diagnosis is essential for better patient outcomes due to prompt treatment in the neonatal period. We report a case of a female infant at 39 weeks of gestation who was delivered from a 32-year-old mother. The infant was in utero diagnosed by ultrasonography with a hepatic mass, most likely hemangioma. The mass was resected after birth and it was diagnosed as infantile hepatic hemangioendothelioma type II. The course of the disease was excellent and the patient was cured after treatment.

## Introduction

Infantile hepatic hemangioendothelioma/hemangioma (IHH) is the most common hepatic tumor of mesenchymal origin [1]. The first encounter was reported by Kunstadter in 1933 [2]. In most studies, IHH is found in infants under six months. It accounts for approximately 1% of all tumors in children. The patient usually demonstrates signs and symptoms before the first year of life [2]. This tumor can be incidentally found in the fetus by ultrasonographic scan during antenatal care of the mother. It can also be found in children who underwent abdominal computed tomography (CT) from other indications. The patients may present with tumor complications such as coagulopathy, heart failure, or jaundice [1]. IHH is divided according to histology into two types [3]. Type I IHH is composed of vascular channels lined with benign endothelial cells displaying small nuclei. Type II IHH demonstrates vascular spaces with pleomorphic endothelial cells with hyperchromatic nuclei and mitosis. Type II IHH was once classified as a high-grade tumor due to its more aggressive histopathological characteristics than type I [3]. However, the histopathological features do not indicate malignant behavior compared to type I IHH [3]. Treatment modalities of IHH show diversity including supportive care [3], and pharmacotherapy such as steroid or interferon [4] or surgical intervention in a large lesion [3]. We presented a case of infantile hepatic hemangioendothelioma type II observed at Maharaj Nakorn Chiang Mai Hospital, along with radiologic and pathologic findings.

## Case presentation

A second child, a female neonate at 39 weeks gestation, with a bodyweight of 3,335 g, was born to a 32-year-old mother with vaginal birth. The pregnancy progressed successfully with no obstetric complications. However, patient history indicated that the mother had 10 years of underlying chronic hepatitis B virus (HBV) infection. She was previously treated with antiviral medication, but she decided to abandon the treatment three years before her first pregnancy. Further antenatal care laboratory tests showed positive HBsAg, positive HBeAg, and a high HBV viral load of 43,525,558 IU/mL. The mother also received tenofovir to prevent perinatal HBV transmission at 27 weeks gestation. Prenatal tests, according to the Thai national antenatal care program, are negative for syphilis and HIV. Prenatal ultrasonography for anomaly screening in the second trimester period was uneventful. However, at the gestational age of 36 weeks, a heterogeneous mass (2.7 x 2.3 x 2.5 cm) in the left lobe of the fetal liver was incidentally discovered by ultrasonography during maternal antenatal care. The lesion revealed a well-circumscribed peripheral vascularization accompanied by a low internal flow, giving the impression of a liver mass, most likely hemangioma.

Physical examination

The neonate weighed 3,335 g with normal activity, and mild pallor without jaundice was observed. The heart sounds were normal without murmurs, and the chest movement was symmetrical. A physical examination of the abdomen indicated no distention with active bowel sounds and non-palpated liver and spleen. No other deformities such as orofacial clefts were identified. In company with a physical examination, no neural tube defects were detected. Lastly, the skin had no cutaneous hemangioma, petechiae, or purpura.

Imaging study and laboratory findings

**Figure 1:**
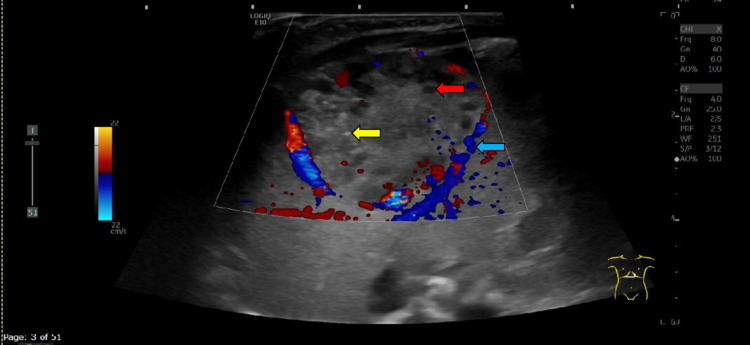
Abdominal ultrasonography for evaluation of the lesion in the left lobe liver. A transverse sonogram demonstrates a well-circumscribed oval-shaped heterogeneously hypoechoic mass with cystic spaces (red arrow), small hyperechoic structures (yellow arrow) representing tiny spots of calcification, and increased peripheral vascularity in the left lobe liver (blue arrow).

When the patient was seven days old, abdominal ultrasonography was performed to evaluate the lesion in the left lobe liver. It showed a normal-sized liver with a well-circumscribed oval-shaped heterogeneously hypoechoic mass measuring 3.75 x 2.82 cm in the left lobe liver. Cystic spaces and tiny calcifications were found in the mass. An increased peripheral vascular flow was discovered. The celiac trunk, hepatic artery, hepatic veins, and portal veins were normal in size. No bile duct dilations were uncovered. All findings indicated that infantile hepatic hemangioendothelioma was possible (Figure 1).

**Figure 2:**
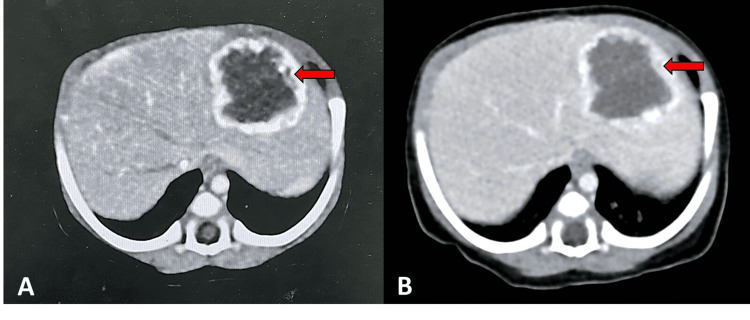
Multidetector CT scan of the upper abdomen using plain CT and dual-phase contrast-enhancement CT. (A) CT in the arterial phase and (B) CT in the portovenous phase demonstrate a hypodense mass with nodular peripheral enhancement in the hepatic segment 2 (red arrow).

A multidetector computed tomography (CT) of the upper abdomen was performed using plain CT and dual-phase contrast-enhancement CT when the patient was 16 days old. CT was performed to fully characterize the mass before treatment decision. A mass was present in the lateral segment of the left lobe of the liver, measuring 3.7 x 4 x 3.2 cm. Stippled calcifications were also noted in the mass. It had avid nodular peripheral arterial enhancement with incomplete centripetal fill-in in the portovenous phase. The celiac trunk and hepatic artery were normal in size. There was no change in the aortic caliber above and below the celiac origin. The attenuation of the enhancing portion equaled that of the aorta in both arterial and venous phases. These findings were compatible with infantile hepatic hemangioendothelioma (Figure 2, panels A and B).

Laboratory investigations were performed on the first day of life, as shown in the table. Complete blood cell count, coagulogram, and liver function test are demonstrated in Table 1. On the fourth day of life, the thyroid function test was performed and the results are shown in Table 2.

**Table 1:** Results of the laboratory investigations. MCV: mean corpuscular volume; MCH: mean corpuscular hemoglobin; MCHC: mean corpuscular hemoglobin concentration; RDW: red cell distribution width; PT: prothrombin time; INR: international normalized ratio; aPTT: activated partial thromboplastin time; AST: aspartate aminotransferase; ALT: alanine aminotransferase; APL: alkaline phosphatase *Reference range is derived from Fish et al., 2021 (Appendix 1) [5] and Engorn and Flerlage, 2015 [6].

Parameter		Value	Reference range*
Complete blood count	Hemoglobin	13.6 g/dL	14.5-22.5 g/dL
	Hematocrit	43.9%	45.0-67.0%
	White blood cell	13,160 cells/cu mm	9,000-30,000 cell/cu mm
	Neutrophils	51.0%	40.0-74.0%
	Eosinophil	2.0%	0.0-7.0 %
	Basophil	0.0%	0.0-2.0%
	Lymphocyte	34.0 %	19.0-48.0%
	Monocyte	9.0%	3.0-9.0%
	Platelet	484,000 cells/cu mm	242,000-378,000 cell/cu mm
	MCV	76.6 fL	95.0-121.0 fL
	MCH	23.7 pg	31.0-37.0 pg
	MCHC	31.0 g/dL	29.0-37.0 g/dL
	RDW	27.9%	11.5-14.4%
Blood coagulogram	PT	10.80 s	10.1-15.9 s
	INR	0.96	0.53-1.62
	aPTT	34.80 s	31.3-54.5 s
	Fibrinogen	419.7 mg/dL	167-399 mg/dL
Liver function test	Total bilirubin	0.88 mg/dL	<8.0 mg/dL
	Direct bilirubin	0.51 mg/dL	<0.6 mg/dL
	Total protein	6.8 g/dL	4.6-7.0 g/dL
	Albumin	3.7 g/dL	3.0-3.9 g/dL
	Globulin	3.1 g/dL	3.1-3.5 g/dL
	AST	62 U/L	47-150 U/L
	ALT	12 U/L	13-45 U/L
	ALP	93 U/L	150-420 U/L

**Table 2:** Results of thyroid function test TSH: thyroid-stimulating hormone; FT4: free thyroxine; FT3: free triiodothyronine *Reference range is derived from the local laboratory reference.

Parameter	Value	Reference range*
TSH	6.7 uIU/mL	0.7-15.2 uIU/mL
FT4	3.32 ng/dL	0.86-2.49 ng/dL
FT3	7.66 pg/mL	1.73-6.3 pg/mL

Patient management

The patient received consultation by a pediatric hematologist on further treatment planning. The Kasabach-Merritt phenomenon was later followed up, and the results were unremarkable. The patient had no symptoms or complications from the suspected infantile hepatic hemangioendothelioma/hemangioma. Wait and watch could be the option in this patient, but complications from progressive tumor should be monitored. Pharmacotherapy could be prescribed to ameliorate the tumor progression. After discussion, the patient's parents refused any medication because they are anxious about the adverse drug reactions. Observational treatment was proposed and monitoring for complications would be followed. However, parents had concerns about the remaining liver tumor and potential transformation. Tissue diagnosis would be explicit for the conclusion. Multidisciplinary team discussion, including pediatric surgeons, was performed to carry out the treatment strategy. After the discussion between the family and the multidisciplinary team, the parents made the decision for the procedure of tumor removal.

No medication was prescribed during the follow-up time, and surgical resection was planned due to the parent’s concern. At three months, the surgeon performed a hepatic segmentectomy to remove liver segment 2 from the patient. Intraoperative findings showed a 2.5 cm diameter mass at segment 2 at the liver with a one-centimeter internal calcification. Neither local invasion nor ascites was seen at the site. Further observations indicated that the gallbladder was unremarkable without bile duct obstructions.

Furthermore, the hepatitis B vaccine and hepatitis B immunoglobulin were prescribed at birth to prevent perinatal HBV transmission for infants born to infected mothers. HBV status will be monitored at the age of one year. In addition, the patient had mild anemia and was later diagnosed with hemoglobin H disease. Folic supplementation was prescribed, and no transfusion was required.

Pathological findings

**Figure 3:**
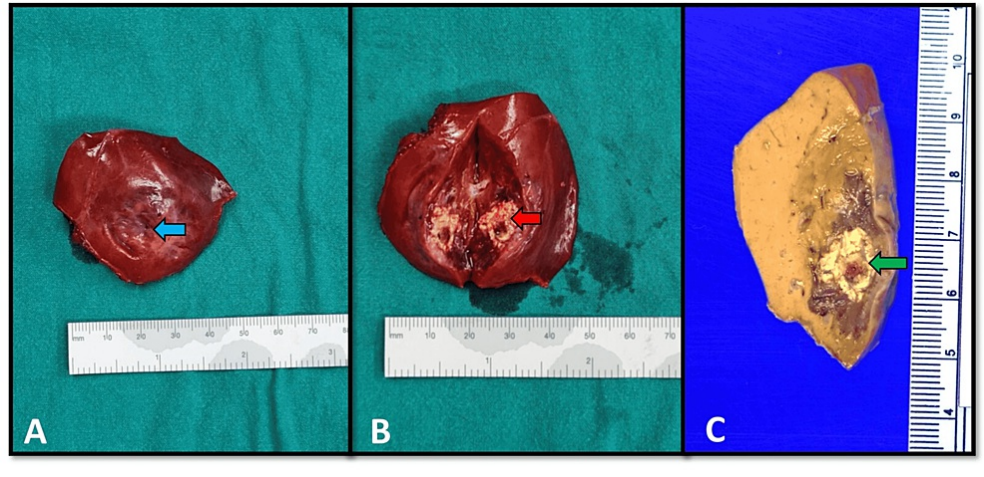
Macroscopic findings of the resected hepatic segment 2. (A) The surgical resection of a hepatic segment 2 shows focal retraction of the hepatic capsular surface (blue arrow). (B) The cut section of the hepatic segmentectomy specimen demonstrates a well-defined bordered light-brown mass with central calcification (red arrow). (C) The formalin-fixed segment of the liver shows a well-defined bordered light-brown mass with central calcification (green arrow). The non-neoplastic hepatic parenchyma is unremarkable.

**Figure 4:**
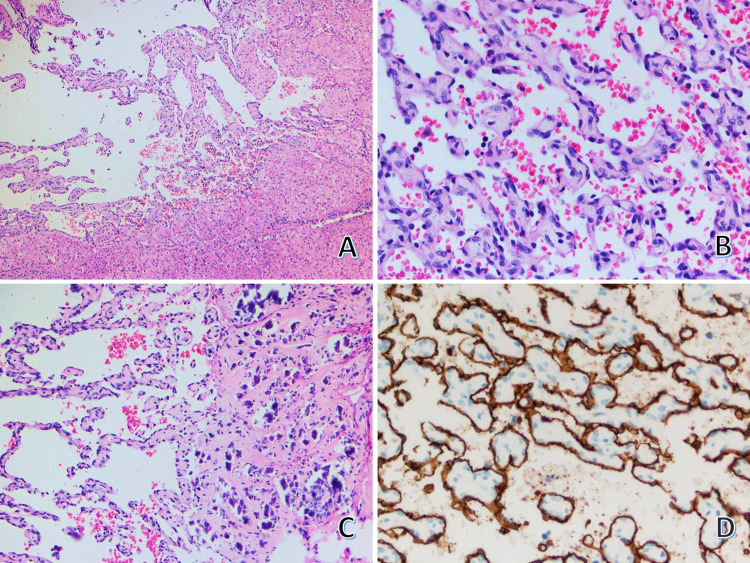
Microscopic findings of the liver with an immunohistochemical stain. (A) Low magnification of the tumor shows irregular vascular channels without capsules. These vascular channels contain red blood cells (H and E, 40×). (B) High magnification of the tumor reveals irregular vascular spaces lined with pleomorphic endothelial cells with hyperchromatic nuclei (H and E, 400×). (C) Microscopic finding of the tumor shows irregular vascular spaces with foci of dystrophic calcification (H and E, 200×). (D) Immunohistochemical stain (CD34) shows positive result in the endothelial cells (400×).

The surgical specimen consisted of a liver measuring 5.3 x 4.8 x 2.5 cm with a focal retraction on a capsular surface and weighing 18 g. Serial sections showed a firm light-brown nodule measuring 3 x 2.8 x 1.8 cm with areas of calcification. The gross pathological findings are illustrated in Figure 3, panels A-C. Microscopically, the nodule revealed vascular channels with an irregular border (Figure 4, panel A). Some vascular channels showed irregular branching spaces. No significant atypia with occasional hobnail appearance of endothelial cells presented with an infrequent mitotic count (Figure 4, panel B). Dystrophic calcification was also noted (Figure 4, panel C). The hepatocytes adjacent to the tumor showed anastomosis between hepatic sinusoids and intratumoral vascular channels. In contrast, the hepatocytes far from the tumors are arranged in a trabecular pattern with one or two cells thick. Bile ductules, hepatic arteries, and portal veins were unremarkable. Immunohistochemical stains revealed that the dilated vascular channels were positive for CD34 (Ventana) in the plump endothelial cells (Figure 4, panel D). These morphologic features were consistent with infantile hepatic hemangioendothelioma type II.

Patient course

The patient was asymptomatic during the follow-up time and laboratory monitoring was uneventful. The liver mass was slightly decreased from follow-up ultrasonography. After surgery, at the age of three months, the patient’s clinical findings gradually improved. She was active with pink skin color. The oxygen saturation in room air was more than 95%. She also had no difficulty in feeding or excreting from the mouth. No surgical complications were later observed, and her parents continued to provide routine care to the baby.

## Discussion

Infantile hepatic hemangioendothelioma/hemangioma (IHH) is a neonate’s common hepatic mesenchymal tumor. Generally, at the point of diagnosis, 90% of patients are less than six months old [1]. The tumor is slightly more predominant in females [2]. Ten to 40% of IHH have coexisting cutaneous cavernous hemangiomas, while 50% of the cases are incidental findings at autopsy. Associated abnormalities accompanying this disease included the following: developmental defects, chromosomal abnormalities, hemangiomas, mesenchymal hamartoma, and Wilms tumor [3]. Some liver tumors in children demonstrate possible etiologies, such as the transmission of maternal hepatitis B virus (HBV) to the fetus for the cause of hepatocellular carcinoma (HCC); however, no evidence that maternal HBV transmission or administration of tenofovir can cause IHH in the neonate [1]. In addition, neonatal abnormalities were not discovered in this case; therefore, the occurrence of IHH is spontaneous.

Patients with IHH sometimes show variation in clinical presentation depending on the tumor size and location. Usually, abdominal distension or palpable hepatic mass is present. Further, cardiac complications such as congestive heart failure and hematologic complications such as thrombocytopenia, anemia, and Kasabach-Merritt syndrome are observed [4,7]. In addition, jaundice, increased hepatic enzymes (aspartate aminotransferase {AST} and alanine aminotransferase {ALT}), consumptive hypothyroidism, difficulty in breathing, failure to thrive, and shock due to tumor rupture may be present in the patients. We found a slight elevation of free thyroxine (FT4) and free triiodothyronine (FT3) levels with normal TSH on the fourth day of life. However, the follow-up thyroid function test was subsequently normal. The transition from the fetal to neonatal period was the explanation for this event. The presence of a thyroid stimulating hormone (TSH) surge in neonates results in increased thyroid hormone synthesis, and the normalization of thyroid hormone may require a longer time compared to TSH level [8]. Serum alpha-fetoprotein (AFP) is beneficial for liver tumor discrimination and elevation of AFP is commonly found in hepatoblastoma, hepatocellular carcinoma, and also secreting germ cell tumors [3,9]. The normal level of AFP is commonly seen in IHH [10]. However, a rising level may be observed in this tumor [11]. The elevation of AFP at initial diagnosis in a neonate with a liver tumor may be misinterpreted due to the physiologic AFP elevation in this age group [12], therefore serial AFP evaluation until normalized should be indicated [9]. Unfortunately, AFP was not evaluated in our case.

Imaging studies of IHH show a space-occupying lesion in the liver. By computed tomographic scan, infantile hemangioendothelioma presents a well-defined border mass alongside decreased attenuation compared to the adjacent non-neoplastic liver tissue [3]. The areas containing hyper-attenuation may be present in the tumors with hemorrhage or calcification. The tumor reveals enhancement figures similar to that of other vascular tumors (e.g., hemangioma) [3].

Histologically, IHH is classified into two types. Type I IHH shows the proliferation of small, capillary-like vascular spaces lined by bland or plump endothelial cells. The vascular channels are separated by connective tissue interspersed with small bile ducts. The mitotic figures are either rare or absent. Malignant spindle cell components are not present. Type II IHH shows disorganized branching vascular channels lined by endothelial cells, which reveal pleomorphism, nuclear hyperchromatism, and frequent mitoses [3]. The endothelial cells in both tumors show positivity with CD31, CD34, and factor VIII-related antigens [11]. The tumor shows branching vascular channels with an infiltrative border lining with endothelium with pleomorphic hyperchromatic nuclei with inconspicuous mitoses in this patient. By using the pathological feature correlating with radiographic findings, all features are consistent with infantile hepatic hemangioendothelioma type II.

The differential diagnosis of infantile hepatic hemangioendothelioma is a cavernous hemangioma, angiosarcoma, hepatoblastoma, and mesenchymal hamartoma [11]. The cavernous hemangioma is the most common benign hepatic tumor arising from vascular malformations [13,14]. Patients are usually asymptomatic, and the lesion is often incidentally found in the imaging study. Ultrasonographic findings show a well-circumscribed homogeneous nodule. Computed tomographic scans with contrast reveal a well-circumscribed hypodense lesion alongside peripheral nodular enhancement [15]. Angiosarcoma is a high-grade malignant hepatic tumor [16]. The tumor shows a diffusely infiltrative mass with dark red cut surfaces due to hemorrhage intermixing with gray-white solid areas and blood-filled spaces. Histopathology comprises infiltrative, freely anastomosing vascular channels lined with endothelial cells showing abundant, pale eosinophilic cytoplasm with hyperchromatic nuclei [17]. Hepatoblastoma is the most common primary malignant hepatic tumor in infants. The patients are usually younger than five years and generally present with a single liver mass with or without small satellite nodules [17]. Sometimes, multinodular hepatic masses with well-defined borders and tan-brown cut surfaces are presented. Alpha-fetoprotein levels increase in more than 90% of the cases of hepatoblastoma [18]. The tumor is classified into several patterns: fetal, embryonal, small cell undifferentiated (SCUD), cholangioplasty, and micro trabecular [19]. Mesenchymal hamartoma is the third most common childhood hepatic tumor. The patients are usually asymptomatic, and alpha-fetoprotein may not increase or increase mildly. The tumor appears as a well-circumscribed mass with cystic cut surfaces. Histopathology shows branching bile ducts embedded in the loose myxomatous stroma mixing with myofibroblast-like cells. Dilatation of small blood and lymphatic vessels is also noted in the lesions [3].

IHH is classified as a benign vascular tumor with spontaneous regression in some patients [3,20]. However, Moon et al. reported that IHH type II can progress into hepatic angiosarcoma [21]. Moreover, factors that indicate that the patient will have a poor prognosis include multiple tumor masses, heart failure, and complications (e.g., Kasabach-Merritt syndrome) [22]. After the first year of life, the tumor may show spontaneous regression. Nonetheless, patients may present complications such as high output heart failure or coagulopathy. The size of the tumor and severity of symptoms determine the treatment modalities. Interventions are helpful in symptomatic cases which fail concerning conservative treatments. Pharmacotherapy with steroid or vincristine has been reported as the first-line treatment for IHH [23]. In addition, beta-blocker, propranolol, can be used as the first-line treatment in patient with the diffuse type of IHH [23]. Besides, there has been reported that the tumor shows rapid regression when adding of interferon (IFN) was made [21]. In asymptomatic patients, wait and watch could be the option, but complications from progressive tumor should be monitored [24]. In the symptomatic patient with medical treatment failure, surgery would be indicated [24]. Surgical resections should be considered in patients with life-threatening symptoms or that the mass cannot be discerned from other malignant tumors. A natural involution of the mass can be stimulated by applying steroids and interferon [25]. In this case, due to the parent's concern, surgical resection was performed to remove the tumor instead of administrating pharmacotherapy and waiting for tumor regression.

Perinatal diagnosis plays a vital role in postpartum care [25]. In this patient, the tumor was detected in utero, which gave the surgeon time to plan the treatment, and, upon birth, the patient promptly received treatment leading to a good prognosis.

## Conclusions

Infantile hepatic hemangioendothelioma is one of the causes of liver mass in neonates. Physical examination, radiographic findings, and pathological findings are essential for the definite diagnosis and differentiation from other hepatic masses. Kasabach-Merritt syndrome and congestive heart failure are probable complications and should be investigated. It is important to note that surgery plays an important role in the case presenting with complications, failure of supportive treatment, or refusal of medical therapy. Prenatal diagnosis is also essential in early diagnosis, with prompt treatment after birth leading to an excellent outcome.
